# Systematic Study of Ciprofloxacin Release from Lipid-Based Nanocarriers

**DOI:** 10.3390/pharmaceutics18060727

**Published:** 2026-06-12

**Authors:** Eva Carolina Arrua, Cintia Briones Nieva, Santiago Nicolás Campos, Andrea Paola Rivas Marquina, Giselle R. Bedogni, Claudia Llanos, Alicia Graciela Cid, Mercedes Villegas, Elio Emilio Gonzo, Claudio Javier Salomon, José María Bermúdez

**Affiliations:** 1 Centro de Investigación y Desarrollo en Materiales Avanzados y Almacenamiento de Energía de Jujuy-CIDMEJu (CONICET-Universidad Nacional de Jujuy), Centro de Desarrollo Tecnológico General Savio, Palpalá 4612, Argentina; arivas@cidmeju.unju.edu.ar; 2 Facultad de Ciencias Exactas, Universidad Nacional de Salta, Av. Bolivia 5150, Salta 4400, Argentina; 3 Instituto de Investigaciones para la Industria Química, Universidad Nacional de Salta—Consejo Nacional de Investigaciones Científicas y Técnicas, Av. Bolivia 5150, Salta 4400, Argentina; brionesncintia@gmail.com (C.B.N.); campossantiago93@gmail.com (S.N.C.); aliciagracielacid@gmail.com (A.G.C.); merchuvillegas@gmail.com (M.V.); josemariabermudez@gmail.com (J.M.B.); 4 Instituto de Química Rosario (IQUIR-CONICET), Rosario 2000, Argentina; gisellebedogni@gmail.com (G.R.B.); csalomon@fbioyf.unr.edu.ar (C.J.S.); 5 Facultad de Ingeniería, Universidad Nacional de Salta, Av. Bolivia 5150, Salta 4400, Argentina; llanosclaudia1507@gmail.com (C.L.); eliogonzo1945@gmail.com (E.E.G.)

**Keywords:** lipid nanoparticles, solid lipid nanoparticles, nanostructured lipid carriers, ciprofloxacin, drug release, dissolution modeling

## Abstract

**Background/Objectives**: Lipid-based nanocarriers have emerged as promising systems for improving the delivery of poorly soluble drugs by enhancing stability, bioavailability, and controlled release. This work aimed to formulate solid lipid nanoparticles (SLN) and nanostructured lipid carriers (NLC) containing ciprofloxacin (CIP) using solvent-free procedures. **Methods**: The systems were extensively characterized using dynamic light scattering (DLS), transmission electron microscopy (TEM), and atomic force microscopy (AFM) to study the nanoparticles in the solid state. Furthermore, in vitro drug release was evaluated, and mathematical modeling was applied to analyze the resulting release kinetics. Additionally, storage stability was assessed at 4 °C and 25 °C over a period of 8 months. **Results**: The results indicated that SLN with an average size of ~50 nm (SLN 50) and NLC with mean diameters of ~25, 50, and 100 nm (NLC 25, NLC 50 and NLC 100 respectively) were successfully prepared. DLS measurements showed narrow particle size distributions (PdI ≤ 0.2) and negative zeta potentials ranging from −3.7 to −7.7 mV. Encapsulation efficiencies were remarkably high for most systems, reaching ~98% for SLN 50, NLC 50, and NLC 100, while the smallest formulation (NLC 25) showed a lower efficiency (~80%). Both TEM and AFM confirmed the formation of spherical nanoscale structures consistent with the sizes determined by DLS. Release studies revealed a strong influence of particle size on kinetics: NLC 25 exhibited rapid release (~95% within 30 min), whereas NLC 100 showed a sustained profile (<20% after 6 h). Dissolution profiles were accurately described by the Lumped-Gonzo kinetic model (R^2^ > 0.98), enabling estimation of dissolution efficiency. **Conclusions**: These findings confirm that lipid-based nanocarriers can be engineered to precisely control CIP release.

## 1. Introduction

The development of efficient drug delivery systems remains a fundamental challenge in pharmaceutical science, particularly for drugs with poor solubility and bioavailability [1]. The exceptional properties of nanomaterials make them particularly relevant for many applications, such as in pharmaceutical technology [2]. In recent years, lipid-based nanomaterials have emerged as promising carriers for drug delivery due to their unique physicochemical properties and biocompatibility. These nanocarriers, including lipid nanoparticles, solid lipid nanoparticles (SLN), nanostructured lipid carriers (NLC), and liposomes, offer several advantages: they can encapsulate both hydrophilic and lipophilic drugs, improve drug solubility and stability, enhance bioavailability, and provide controlled or targeted release profiles [3,4]. Furthermore, lipid-based systems often reduce systemic toxicity and are more readily accepted by regulatory agencies due to their composition from generally recognized as safe excipients [3].

Among the many considerations in optimizing lipid nanocarrier systems, understanding and predicting drug dissolution behavior is of particular importance. Dissolution is a critical step in the drug absorption process, especially for oral delivery routes, where it influences the rate and extent of drug availability in systemic circulation [5]. Consequently, modeling the dissolution profile of drugs from nanocarriers has become an essential tool in the development of formulations [6]. Such models enable the prediction of release kinetics under various physiological conditions, support rational design decisions, and can reduce the number of costly and time-consuming in vivo experiments. Mathematical modeling approaches, ranging from empirical to mechanistic models, can provide valuable insights into the interplay between drug properties, carrier composition, and environmental factors that influence dissolution performance [7,8,9].

Ciprofloxacin (CIP), a broad-spectrum fluoroquinolone antibiotic, is frequently used as a model drug in dissolution and release studies due to its well-characterized physicochemical properties and clinical relevance [10]. It is almost insoluble in water and its bioavailability is significantly limited by its very low solubility at neutral pH [11]. It is important to highlight that CIP is a class IV drug according to the Biopharmaceutics Classification System [12] due to its poor permeability across biological membranes [13]. CIP exhibits pH-dependent solubility and permeability characteristics, making it a suitable candidate for exploring the effects of formulation strategies on drug release and bioavailability [14]. As reported, several strategies have been applied to overcome such drawbacks using lipid nanocarriers [15,16,17,18,19,20]. However, to the best of our knowledge, the application of mathematical models to evaluate CIP release has not been systematically addressed.

In this context, the present study evaluates the effect of nanocarriers in the delivery of CIP in order to control drug release. For this purpose, CIP SLN and CIP NLC were developed, physicochemically characterized, and compared regarding their drug release behavior. Furthermore, in vitro release profiles were adjusted using the Lumped-Gonzo model and parameters of pharmaceutical relevance were determined. By integrating experimental characterization and mathematical modeling, this study aims to contribute to the rational design of advanced nanocarrier systems with improved physicochemical and release properties while minimizing formulation-related limitations.

## 2. Materials and Methods

### 2.1. Materials

Ciprofloxacin hydrochloride was obtained from Parafarm^®^ (Buenos Aires, Argentina). Kolliphor^®^ HS 15 was obtained from BASF SE (Ludwigshafen, Germany) and soybean lecithin (phosphoglyceride compound consisting of phosphoric acid, fatty acids, glycerol, glycolipids, triglycerides and phospholipids) was provided by Cargill (Minneapolis, MN, USA). Labrafac^®^ PG (propylene glycol esters of caprylic and capric acids) and Gelucire^®^ 44/14 (polyethylene glycol-32 mono- and diesters of lauric acid) were purchased from Gattefossé S.A. (Saint-Priest, France). All the other reagents and solvents used in this study were of analytical grade.

### 2.2. Methods

#### 2.2.1. Formulation of Lipid-Based Nanocarriers

A hot-melt emulsification process was employed to form SLN, based on Islam et al. [21]. Briefly, a lipid phase containing 1200 mg of Gelucire^®^ 44/14, 188 mg of Tween 80, and 52 mg of Span 80 was melted in an oil bath at 70 °C under stirring. The lipid phase was then mixed with 2560 mg of water at 70 °C under vigorous stirring for 5 min. Finally, a cold dilution was carried out by immediately adding 60 mL of water at 4 °C. For drug-loaded SLN, 20 mg of CIP were added into the lipid phase (with Gelucire 44/14, Tween 80 and Span 80) before heating to 70 °C.

To prepare NLCs a phase inversion technique was employed based on previous studies [22], using different quantities of the same components: Kolliphor^®^ HS 15 (846, 1983 and 484 mg), soy lecithin (75 mg), Labrafac^®^ PG (1028, 846 and 1209 mg), NaCl (156 mg), and water (2962, 4055 and 3143 mg) to obtain NLC 50, NLC 25 and NLC 100, respectively. The mixtures were stirred and subjected to three cycles of gradual heating and cooling between 50 and 85 °C. During the last cycle, an irreversible phase inversion was triggered by introducing cold water (4 °C). For drug-loaded formulations, 30 mg of CIP was previously dissolved in Labrafac^®^ PG before proceeding with the described process.

#### 2.2.2. Lipid-Based Nanocarriers Characterization

Particle size (Z-average) and polydispersity index (PdI) were determined through dynamic light scattering (DLS) using a Nano Particle Analyzer SZ-100 (HORIBA Instruments Inc., Irvine, CA, USA). SLN samples were diluted 10-fold, while all NLC samples were diluted 60-fold, both with distilled water. Measurements were carried out at 25 °C in triplicate.

#### 2.2.3. Lipid-Based Nanocarriers Encapsulation Efficiency

Drug encapsulation efficiency of CIP into SLNs and NLCs was determined by an indirect method. First, total nanocarrier solutions were diluted in methanol to dissolve the lipid carriers. Then, an ultrafiltration-centrifugation procedure was used with Amicon^®^ Ultra-4 centrifugal devices with molecular weight cut-off of 100 kDa filters (Millipore, Darmstadt, Germany) to separate excess CIP and residual components from the lipid formulations. Post filtrate samples were prepared by dissolving an exact amount in methanol. The drug concentration and excess of CIP were determined by UV spectrophotometry at 276 nm (Jenway Technology Company, 7315 UV-vis Spectrophotometer, Staffordshire, UK), based on previously published methodologies for similar pharmaceutical systems [23,24,25]. In addition, blank formulations containing all excipients but no ciprofloxacin exhibited negligible absorbance at 276 nm, indicating that the formulation components did not significantly interfere with ciprofloxacin quantification. The UV–Vis spectrophotometric method used for CIP quantification was evaluated in terms of linearity, sensitivity, and precision. Calibration curves in the concentration range of 1.27–7.61 µg/mL showed excellent linearity (R^2^ = 0.9998). The limit of detection (LOD) and limit of quantification (LOQ), calculated according to ICH recommendations, were 0.15 µg/mL and 0.44 µg/mL, respectively. Replicate measurements demonstrated good precision, with low variability among absorbance determinations.

Equation (1) was used to calculate CIP encapsulation efficiency ($E_{ef}$).

(1) $$E_{ef}=\frac{mg\;of\;CIP\;total-mg\;of\;CIP\;outside\;nanocarriers}{mg\;of\;CIP\;total}$$

#### 2.2.4. Morphological Characterization of Lipid-Based Nanocarriers by Transmission Electron Microscopy

Morphology of the SLNs and NLCs was evaluated by transmission electron microscopy (TEM) using a Jeol 100CX instrument (JEOL-France, Paris, France). Sample preparation consisted of 1:60 dilution for SLN and 1:360 dilution for NLC, in order to obtain a better image with separated particles. Samples were stained with an aqueous solution of 0.5% phosphotungstic acid for 2 min, washed with water, dried at room temperature, sprayed onto copper grids overlayed with a 1% solution in chloroform (Mikross, Paris, France), and observed under the microscope.

#### 2.2.5. Atomic Force Microscope Analysis of Lipid-Based Nanocarriers

Atomic force microscope (AFM) was employed to characterize lipid-based nanocarrier surface properties, confirm the results obtained in DLS experiments and evaluate the aggregation behavior of nanocarriers. Fresh lipid-based nanocarriers (SLNs and NLCs) were prepared for each AFM observation stage. Aliquots of 100 µL were washed with 1 mL of ultrapure water by centrifugation (3 cycles at 20,000 rpm for 1 h). After the last washing step, the pellets were resuspended in 0.5 mL of ultrapure water. Subsequently, 5 µL aliquots of each sample were placed on clean glass slides and gently covered with a coverslip to ensure homogeneous distribution of the suspension. Samples were maintained in a clean, humidity-free environment at 25 °C until complete water evaporation. Dried samples on the coverslips were analyzed by AFM using a Keysight 5500 AFM system (Keysight Technologies, Santa Clara, CA, USA) under ambient air conditions. Topographic images were obtained in tapping mode using a commercial Point Probe Plus/Non-Contact/Long Cantilever (Keysight, PPP NLC, N9621-60003, Böblingen, Germany) with a nominal force constant of 48 N m^−1^ and a resonance frequency of 190 kHz. Image analysis was performed using Gwyddion software version 2.68 [26].

#### 2.2.6. Evaluation of Ciprofloxacin Release from Lipid-Based Nanocarriers

In vitro release studies were performed in simulated gastric fluid (pH 1.2, HCl 0.1 N) at 37 °C using the dialysis bag method. The dialysis bag method employed in this study has been extensively reported for ciprofloxacin release evaluation in nanoparticle and drug delivery systems, supporting the suitability of the membrane for diffusion studies without significant drug–membrane interaction [27,28,29,30]. To achieve this, an equivalent of 5 mg of CIP of each nanocarrier was placed inside pre-hydrated dialysis bags (with a cutoff of 12,000–14,000 Da) containing 10 mL of medium. The bags were immersed in a beaker with 100 mL of simulated gastric fluid. The beaker was kept in a thermostatic shaker at 37 °C under stirring at 50 rpm. At predetermined time intervals (0, 30, 60, 120, 180, 240, 300 and 360 min), 1 mL of the release medium was withdrawn, and CIP concentration was determined by absorbance at 276 nm (Jenway 7315 UV-vis Spectrophotometer, Staffordshire, UK), using CIP calibration curves in HCl 0.1 N. All release experiments were performed in triplicate (three independent experiments), and results are expressed as mean ± SD (n = 9). During sampling, 1 mL aliquots were withdrawn from the release medium at each time point without medium replacement. Cumulative CIP release values were corrected considering the progressive reduction in the total release volume throughout the experiment.

#### 2.2.7. Mathematical Modeling of Ciprofloxacin Release

The release behavior of CIP from both lipid-based nanocarriers was analyzed using the Lumped-Gonzo model (Equation (2)), which was developed and validated by our research group [8,9,31,32,33,34,35].

(2) $$M_{t}\%=\frac{a\times t}{\left(1+b\times t\right)}$$

where $M_{t}\%$ is the percentage of drug released at time *t*, *a* ($\%{min}^{-1}$) and *b* (${min}^{-1}$) are the Lumped-Gonzo model representative parameters.

Using this model, parameters of pharmaceutical relevance were determined to compare the different CIP release profiles, including release time ($t_{x\%}$), percentage of drug released (X%*_t_*), dissolution efficiency (DE), and mean dissolution time (${MDT}_{x\%}$).

The $t_{x\%}$ is the time required to reach a given amount of released CIP. The X%*_t_* is the percentage of drug released at a certain time. The DE is defined as the area under the dissolution curve up to a certain time, expressed as a percentage of the area of the rectangle described by 100% dissolution at the same time (Equation (3)) [36].

(3) $$DE=\left(\frac{\int_{0}^{t_{F}}M_{t}\%\times dt}{100\times t_{F}}\right)*100$$

On the other hand, the ${MDT}_{x\%}$ is the mean time required for release drug to a certain point under experimental conditions (Equation (4)).

(4) $${MDT}_{X\%\;}=\frac{\sum_{j=1}^{n}t_{jm}\times ∆M\%}{\sum_{j=1}^{n}∆M\%}$$

where $t_{jm}=\frac{\left(t_{j}+t_{j-1}\right)}{2}$ is the average time between two points and ∆M% is the additional amount of drug released between $t_{j}$ and $t_{j-1}$.

According to the model equation (Equation (2)), DE, $t_{x\%}$ and ${MDT}_{X\%\;}$ are given by Equation (5), Equation (6) and Equation (7), respectively.

(5) $$DE=\left(\frac{a}{b^{2}}\right)\;\frac{\left[b\times t_{F}-ln(1+b\times t_{F})\right]}{100\times t_{F}}*100$$

(6) $$t_{X\%}=\frac{X\%}{\left(a-b\times X\%\right)}$$

(7) $${MDT}_{X\%}=\frac{a}{b^{2}}\;\frac{\left[ln\left(1+b\times t_{X\%}\right)-\frac{b\times t_{X\%}}{\left(1+b\times t_{X\%}\right)}\right]}{M\%(t_{X\%})}$$

#### 2.2.8. Storage Stability of Lipid-Based Nanocarriers

The stability study was conducted using the CIP-loaded formulations stored at their original preparation concentration under two storage conditions: at room temperature (25 °C) and at refrigeration temperature (4 °C) for a period of 8 months. For each lipid-based nanocarrier, size, PDI, and zeta potential (ZP) were evaluated. For this purpose, at different times (7, 14, 28, 31, 60, 90, 120, 150, 180, 210 and 240 days) a sample was withdrawn and diluted appropriately for DLS characterization, as detailed in Section 2.2.2.

#### 2.2.9. Statistical Analysis

All obtained results are shown as mean values ± SD. Regression and statistical analysis were performed in Sigma Plot 12.0 software (Systat Software Inc., London, UK). For statistical evaluations, a *p*-value less than 0.05 (*p* < 0.05) was considered significant.

## 3. Results and Discussion

### 3.1. Formulation and Physicochemical Characterization of Lipid-Based Nanocarriers

Two different methodologies were evaluated to formulate lipid-based nanocarriers; a hot-melt emulsification process to obtain SLNs, and a phase inversion technique for NLCs. Additionally, to obtain different NLCs, the proportions of the same reagents were varied. It should be emphasized that both methodologies are carried out entirely without the use of organic solvents, thereby avoiding potential issues related to toxicity, environmental impact, and regulatory restrictions. Furthermore, the process involves exclusively excipients that are not only pharmaceutically acceptable but also officially recognized as safe for human use, which ensures compliance with established safety guidelines and enhances the suitability of the approach for pharmaceutical applications. Table 1 summarizes particle sizes (Z-average), PdI and ZP, determined by DLS, and $E_{ef}$ of all nanocarriers with and without CIP. The measurements revealed that the obtained formulations exhibited well-defined hydrodynamic diameters, SLN with mean values of approximately 50.7 nm and 50.1 nm without and with CIP; while NLCs showed sizes of: 24.9 nm and 25.1 nm, 49.2 nm and 48.9 nm, 119.6 nm and 107 nm, without and with CIP, according to the proportions of reagents utilized. The particle size distribution profile of the analyzed CIP-loaded lipid-based nanocarriers is provided in Figure S1 (Supplementary Materials), which illustrates in detail the overall dispersion pattern of the systems.

Importantly, the analysis demonstrated that all formulations presented a monomodal particle size distribution characterized by remarkably low PdI values (≤0.21 in all cases). These findings provide strong evidence for the high degree of homogeneity achieved in the nanocapsule suspensions, since, as generally acknowledged, PdI values below 0.3 are indicative of a narrow and uniform particle size distribution within nanoscale systems [37]. It is worth noting that no significant differences were observed in the PdI values when comparing non-loaded and CIP-loaded nanocarriers. A slight reduction in the average particle size was observed for some CIP-loaded formulations compared to the corresponding unloaded systems (Table 1). Although these differences were relatively small, they may be associated with interactions between CIP and the lipid/surfactant matrix during nanoparticle formation. Due to its amphiphilic nature, CIP may interact with polar surfactant head groups while partially partitioning into the lipid domains, promoting a more compact interfacial organization. In addition, the presence of CIP during cooling and solidification could slightly influence lipid packing and nanoparticle assembly, contributing to subtle variations in hydrodynamic diameter. This result indicates that the incorporation of the drug did not alter the overall size distribution profile of the systems, thereby suggesting that the structural uniformity and homogeneity of the nanocarriers were preserved regardless of the presence or absence of CIP.

Regarding surface properties, the formulations were found to have a negative zeta potential, with values ranging from −7.7 to −3.7 mV. Such negative surface charges are consistent with those typically reported for similar lipid-based nanocarriers [21,38], thus supporting the reproducibility of the preparation method and its alignment with previously described systems. The comparison with unloaded nanocarriers revealed that the incorporation of CIP did not generate variations in surface charge, as shown in Table 1.

Concerning the NLCs, a direct relationship was observed in the amount of Labrafac^®^ PG included in the formulation and the final particle size: the smallest nanocapsules corresponded to the lowest proportion of Labrafac^®^ PG, whereas the largest particles were obtained when the highest oil content was employed. These observations are fully consistent with previous studies, which have shown that the oil phase concentration is directly correlated with the mean diameter of lipid nanocapsules [22,38]. Taken together, these results confirm that both the physicochemical characteristics and the composition of the formulations meet expectations for homogeneous lipid-based nanocarrier systems.

Regarding the encapsulation process, it must be underlined that the amount of CIP effectively incorporated into the lipid-based nanocarriers is strongly influenced not only by the intrinsic physicochemical properties of the active compound itself, but also by the specific methodology employed during the preparation. The $E_{ef}$ values obtained for the different particle sizes were notably high, reaching 97.9% for SLN, 80.4%, 98.0% and 98.7% for NLC of 25, 50 and 100 nm, respectively. A comparison between the two nanocarrier systems of equivalent particle size revealed that their $E_{ef}$ were essentially comparable, indicating, in this case, that the size of the carrier itself does not play a decisive role in determining the efficiency of drug entrapment under the conditions employed. According to the results of NLCs, when the amount of Labrafac^®^ PG was increased, the $E_{ef}$ was higher (Table 1). These results are consistent with previously reported $E_{ef}$ values for other drugs encapsulated within lipid-based nanocarriers of similar composition and design [21,38,39], further supporting the robustness and reproducibility of the technique used.

### 3.2. Morphological Characterization of Lipid-Based Nanocarriers by Transmission Electron Microscopy

**Figure 1:**
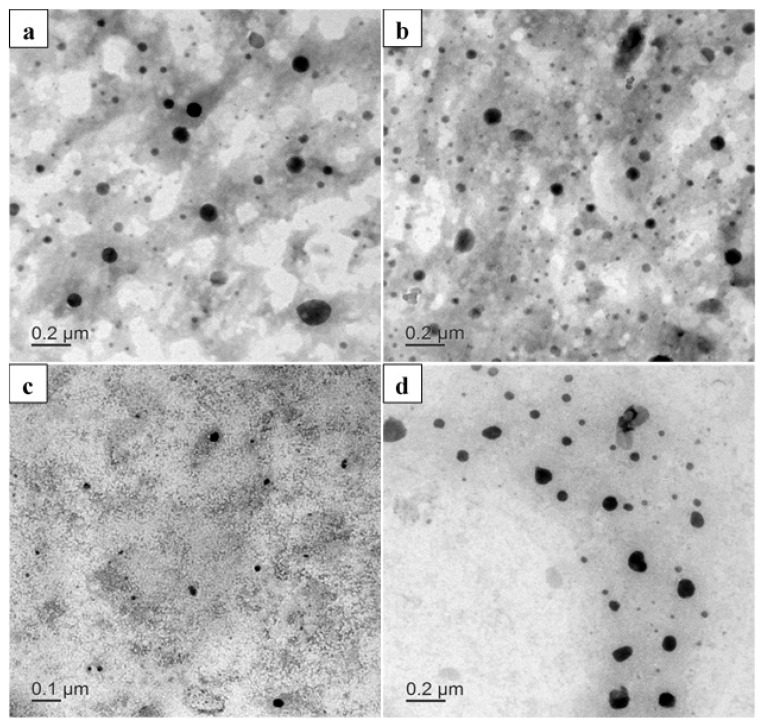
Transmission electron micrographs of lipid-based nanocarriers, SLN 50 (**a**), NLC 50 (**b**), NLC 25 (**c**) and NLC 100 (**d**).

TEM was employed to observe the morphology of the prepared nanostructured systems. The obtained images revealed that all lipid-based nanocarrier formulations exhibited a well-defined and predominantly spherical morphology (Figure 1), which is in agreement with previously reported observations of similar lipid nanocarriers in the literature [22,40,41,42]. The particle dimensions estimated by TEM matched those measured by DLS, validating the accuracy of the particle size determination methods.

A more detailed examination of the micrographs also made it possible to identify qualitative differences among the formulations. The SLN formulation with a nominal size of 50 nm (Figure 1a) showed discrete, dark spherical structures uniformly distributed over the grid, suggesting a relatively homogeneous population of particles. Similarly, the NLC formulation with a size of 50 nm (Figure 1b) displayed spherical particles with comparable contrast and dispersion, although a slightly wider distribution of particle diameters could be visually appreciated, which is consistent with the typically more heterogeneous internal structure reported for NLC systems.

For NLC 25 formulation (Figure 1c), very small, finely dispersed particles were observed distributed throughout the background matrix. The reduced particle diameter made individual particles appear less contrasted but clearly distinguishable, confirming the successful preparation of nanosystems in the smaller size range. In contrast, the NLC 100 formulation (Figure 1d) exhibited larger structures with increased electron density and a tendency toward localized clustering, which may reflect the larger particle size and possible partial aggregation during sample preparation or drying on the TEM grid.

Overall, the TEM observations corroborate the nanoscale dimensions, spherical morphology, and acceptable dispersion of the developed lipid nanocarriers, supporting the suitability of the preparation method for obtaining SLN and NLC systems with controlled particle sizes.

### 3.3. Atomic Force Microscope Analysis of Lipid-Based Nanocarriers

**Figure 2:**
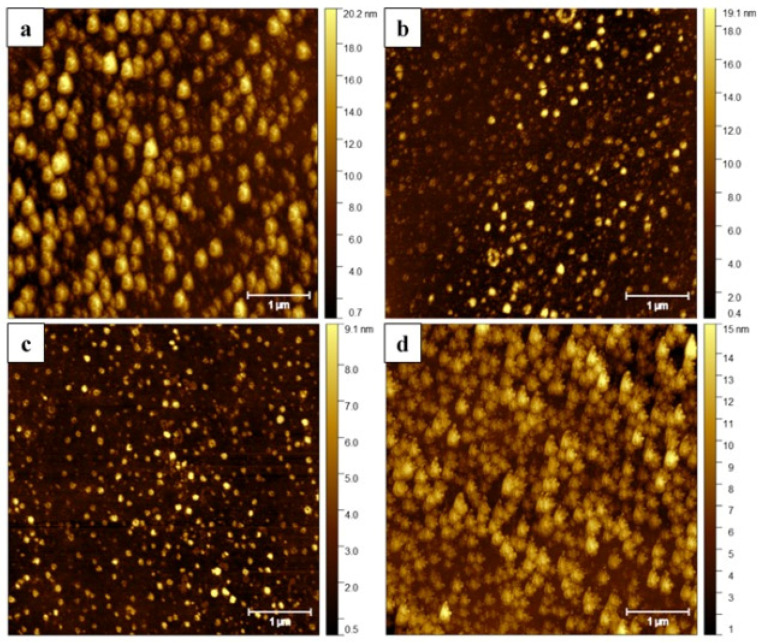
AFM images of lipid-based nanocarriers, SLN 50 (**a**), NLC 50 (**b**), NLC 25 (**c**) and NLC 100 (**d**) in water.

Atomic force microscopy (AFM) was employed to investigate the surface morphology and topographical features of the lipid-based nanocarriers. Representative images of SLN 50 (a), NLC 50 (b), NLC 25 (c), and NLC 100 (d) are shown in Figure 2. In all cases, nanoscale structures were observed distributed across the substrate, confirming the successful formation of lipid-based nanocarriers, in agreement with previous reports [43,44,45,46].

Quantitative analysis of more than 50 particles per formulation revealed average equivalent diameters of 88 ± 25 nm (n = 105) for SLN 50, 269 ± 55 nm (n = 68) for NLC 50, 80 ± 16 nm (n = 133) for NLC 25, and 337 ± 108 nm (n = 51) for NLC 100 (Figure S2). These values are larger than those obtained by DLS (Table 1), which can be attributed to the different measurement principles of each technique. While DLS provides the hydrodynamic diameter of particles in dispersion, AFM measures dried samples on a substrate, where aggregation and deformation effects may occur [47,48,49].

Indeed, the AFM images suggest that the observed structures correspond to aggregates of smaller primary nanoparticles rather than isolated particles. This aggregation is commonly reported for nanoparticle systems during sample drying, where interparticle interactions and the removal of stabilizing agents promote clustering [46,48,49]. Additionally, soft lipid nanoparticles are particularly susceptible to deformation and restructuring upon deposition, which can further contribute to the observed size discrepancies [43,50].

The measured particle heights were significantly lower than the lateral dimensions, with average values of 14 ± 2 nm (SLN 50), 11 ± 2 nm (NLC 50), 8 ± 1 nm (NLC 25), and 8.0 ± 0.7 nm (NLC 100). This difference is consistent with the flattening of soft lipid-based nanoparticles on solid substrates, leading to a “spherical cap” geometry due to surface interactions [51,52].

Although both TEM and AFM analyses were performed under dry-state conditions, important differences between the techniques must be considered when interpreting particle dimensions. In TEM, the highly diluted dispersions deposited onto carbon-coated grids allowed visualization of relatively isolated nanoparticles with minimal aggregation, resulting in particle sizes close to the hydrodynamic diameters obtained by DLS. In contrast, AFM samples were pre-washed and prepared by solvent evaporation on glass substrates, a process that may promote aggregation, spreading, and deformation of the soft lipid nanoparticles due to capillary forces and surface interactions during drying. Consequently, AFM predominantly detected larger clustered structures and flattened morphologies, leading to larger lateral dimensions compared to TEM and DLS measurements.

Despite these limitations, AFM analysis supports the nanoscale nature of the developed systems and provides complementary information to DLS and TEM regarding morphology, aggregation state, and surface organization. These observations are consistent with the overall physicochemical characterization and confirm the successful formation of lipid-based nanocarriers.

### 3.4. Evaluation and Mathematical Modeling of Ciprofloxacin Release from Lipid-Based Nanocarriers

In vitro dissolution testing remains an essential approach to evaluate the ability of pharmaceutical systems to release an active compound under physiologically relevant conditions. These assays are not only useful for comparing the behavior of different formulations, but also for anticipating their in vivo performance. Dissolution profiles, which describe the relationship between drug concentration and time, provide key information regarding the characteristics of drug release.

**Figure 3:**
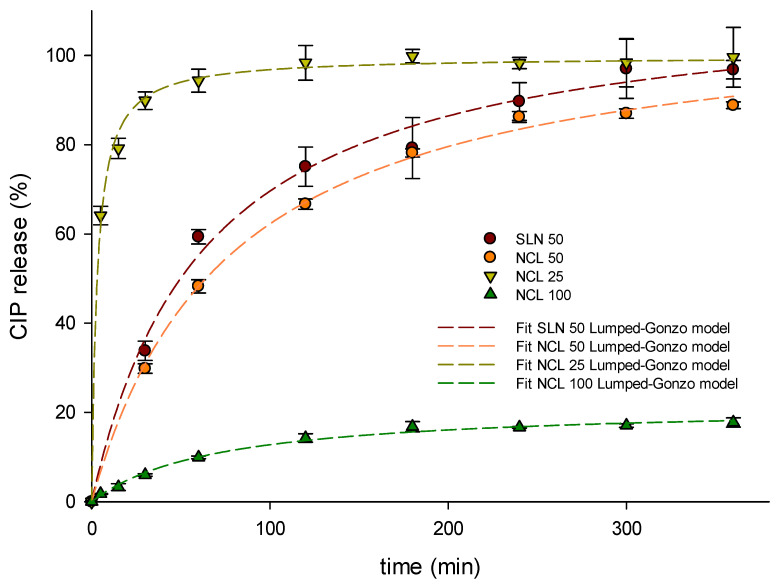
Cumulative percentage of CIP released from lipid-based nanocarriers. Symbols are the mean value of the experimental data and dotted lines represent the Lumped-Gonzo model fitting. All measurements were conducted in triplicates and data is represented as mean ± SD of n = 9, from three independent experiments.

In the present work, the release of CIP encapsulated in lipid-based nanocarriers was evaluated over a 6 h period. The dissolution experiments were designed as a comparative in vitro evaluation under standardized acidic conditions rather than as a direct simulation of the complete gastrointestinal transit. The prolonged assay time was selected to enable full characterization and mathematical modeling of the release profiles, particularly for sustained-release formulations. An observation of the experimental profiles revealed relatively low differences between SLN 50 and NLC 50, since both formulations exhibited almost complete drug release (>90%) after 6 h (Figure 3).

These results contrast with those obtained for nanocarriers of different sizes. Notably, NLC 25 exhibited a significantly faster and nearly complete drug release during the assay. In contrast, larger nanocarriers (NLC 100) showed a substantially lower extent of drug release. This behavior is consistent with previous studies showing that reduction in particle size leads to an increased surface area and shorter diffusion pathways, thereby accelerating drug release [53,54]. Furthermore, it agrees with experimental observations reported for CIP-loaded lipid nanocarriers, where sustained release profiles over extended periods (e.g., ~80% over 10 h) have been associated with larger particle sizes and stronger drug retention within the lipid matrix [54,55]. This tunable release behavior underscores the significant potential of the developed lipid-based nanocarriers as versatile drug delivery platforms. The rapid and nearly complete release exhibited by NLC 25 is particularly suitable for therapeutic scenarios requiring an immediate pharmacological response, whereas the sustained release profile of NLC 100 enables prolonged drug availability, which may improve therapeutic efficacy and patient adherence. These findings demonstrate that precise control of particle size provides a straightforward and effective strategy to tailor drug release profiles according to specific clinical requirements.

The CIP release profiles from the different formulations were fitted using the Lumped-Gonzo mathematical model. This model, based on second-order kinetics, integrates multiple transport phenomena involved in the release process. When this model was applied to CIP-loaded nanocarriers, it demonstrated an excellent ability to fit the experimental data across the entire profile (Figure 3).

As shown in Table 2, which presents the fitting parameters of the Lumped-Gonzo model, the parameter a, associated with the initial release rate, increases as the size of the NLC particles decreases. In contrast, this parameter shows similar values for the SLN and NLC formulations of 50 nm diameter, in accordance with the initial observation of the profiles.

Using the Lumped-Gonzo model, it was possible to determine pharmaceutically relevant parameters such as $t_{x\%}$, ${X\%}_{t}$, DE and MDT to understand and compare release profiles (Table 3).

The ${X\%}_{30\min}$ value was determined to classify the behavior of the formulations according to pharmacopoeia criteria, which state that an immediate-release dosage form must release at least 80% of the drug within that time interval. Based on this criterion, only NLC 25 can be considered an immediate-release system, while the remaining formulations exhibit delayed-release profiles, with significantly lower values.

Regarding ${DE}_{360\min}$, a marked dependence on particle size was observed. Larger diameter formulations exhibited a very low DE, suggesting incomplete and sustained drug release, possibly associated with greater diffusional resistance. In contrast, the 50 nm formulations (both SLN and NLC) showed considerably higher values, indicating intermediate but efficient performance in terms of overall drug release.

These modest differences can be attributed to variations in matrix composition and internal lipid organization, which are known to influence drug diffusion and release kinetics in lipid nanoparticles. In particular, NLCs, composed of mixtures of solid and liquid lipids, may exhibit less ordered internal structures compared to SLNs, affecting drug distribution and release [53].

Finally, ${MDT}_{15\%}$ and $t_{15\%}$ values reinforced these observations. The NLC 25 particles exhibited extremely low values, demonstrating virtually immediate release, while the larger particles showed a progressive increase in these parameters, reaching their maximum values for the NLC 100 formulation. This behavior is consistent with the previously discussed findings and confirms the determining influence of particle size on release kinetics.

### 3.5. Storage Stability of Lipid-Based Nanocarriers

**Figure 4:**
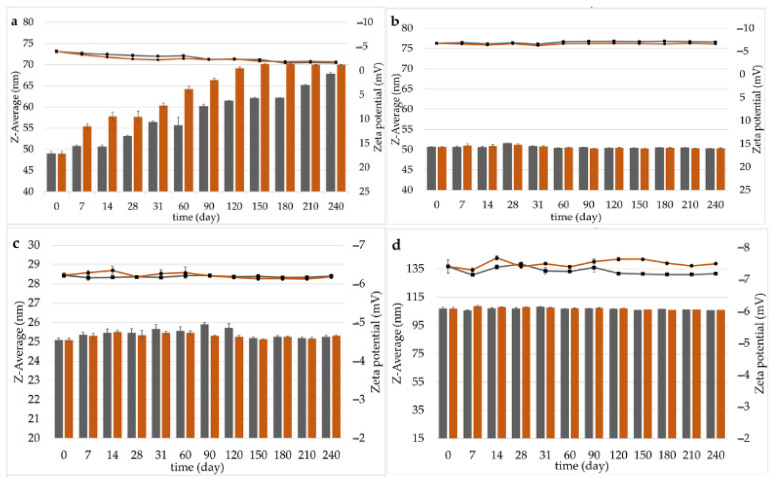
Z-average (nm, bars) and ZP (mV, symbol) changes in SLN 50 (**a**), NLC 50 (**b**), NLC 25 (**c**) and NLC 100 (**d**) at different storage conditions: 4 °C (grey) and 25 °C (orange). All measurements were conducted in triplicates and data is represented as mean ± SD.

The storage stability of the different lipid-based systems was evaluated at two different temperatures, refrigerated at 4 °C and at room temperature (25 °C), for a period of eight months. At predetermined time intervals, aliquots from each developed system were collected and analyzed by DLS. Figure 4 shows the evolution of particle size (Z-average) and ZP of the systems at both temperatures studied.

After 7 days, particle size of SLN 50 (Figure 4a) showed significant changes under both storage conditions, being more pronounced when the storage temperature was set at 25 °C. In addition, changes in the PdI of the systems were also evaluated during the eight-month storage period, and the corresponding results are summarized in Figure S3 (Supplementary Materials). Again, SLN 50 exhibited the most significant fluctuations in PdI, indicating a greater instability.

Regarding physical stability, the remaining nanocarrier formulations (all NLC, see in Figure 4b–d) showed only marginal variations in PdI during the eight-month storage period at both temperatures tested. The absence of pronounced increases in PdI indicates that no significant aggregation, melting, or broadening of the particle size distribution occurred over time.

Although slight fluctuations were detected, these changes remained within a narrow range and can be attributed to minor interfacial rearrangements rather than structural destabilization of the systems. Importantly, comparable PdI profiles were observed under refrigerated and room temperature conditions, suggesting that temperature within this range did not critically affect colloidal organization.

Taken together, these results indicate that the structural integrity and colloidal stability of the NLC formulations were largely preserved throughout storage. Despite the relatively low ZP values of the NLC systems (Table 1), their stability can be primarily attributed to steric stabilization mechanisms provided by the non-ionic surfactants present in the formulation. In particular, Kolliphor^®^ HS 15 forms a hydrated poly(ethylene glycol) (PEG) corona at the particle interface, generating a steric barrier that prevents close particle–particle approach and aggregation. In addition, soy lecithin contributes to interfacial stabilization by forming a structured phospholipid layer, which enhances membrane cohesion and reduces interfacial tension. The combined effect of these components results in effective steric hindrance and interfacial stabilization, compensating for the low electrostatic repulsion indicated by the ZP values. The maintenance of a low and stable PdI over time is particularly relevant for pharmaceutical applications, as it reflects dispersion homogeneity and suggests consistent in vitro and in vivo performance.

Overall, this work demonstrates that lipid-based nanocarriers can be rationally designed to achieve tunable ciprofloxacin release profiles by controlling key structural parameters such as particle size. The combined experimental and modeling approach provides a robust framework for the quantitative interpretation of release kinetics and supports the rational design of these systems as a foundational step toward more advanced biopharmaceutical evaluation.

## 4. Conclusions

This study demonstrates the successful formulation of CIP-loaded lipid-based nanocarriers using solvent-free preparation methods, resulting in nanosystems with controlled size, narrow particle size distribution, and high encapsulation efficiency. Both SLN and NLC formulations exhibited spherical morphology and physicochemical characteristics consistent with stable lipid nanocarrier systems.

The results clearly show that nanoparticle size plays a decisive role in CIP release kinetics. The smallest nanocarrier (NLC 25) favoured rapid drug release due to its larger surface area and reduced diffusion pathways, whereas the largest particles (NLC 100) led to a remarkably sustained release profile. Intermediate behavior was observed for SLN 50 and NLC 50, indicating that both formulation composition and particle size contribute to modulating drug release.

Mathematical fitting using the Lumped-Gonzo model provided excellent adjustment of the dissolution profiles and enabled the determination of key pharmaceutical parameters. These results confirm the value of mathematical modeling as a powerful tool for quantitatively interpreting release kinetics and guiding formulation optimization.

Additionally, stability studies indicated that NLC formulations maintained their colloidal integrity over prolonged storage periods, suggesting their potential applicability as stable lipid-based drug delivery systems with tunable release characteristics.

Overall, the combined experimental and modeling approach presented in this work provides valuable information for the design of lipid-based nanocarriers with tunable release profiles and offers a rational framework for the development of drug delivery systems targeting poorly soluble compounds such as CIP.

## Figures and Tables

**Table 1:** Characterization of lipid-based nanocarriers.

	Nanocarrier	Z-Average	PdI	ZP (mV)	$E_{ef}$(%)
− CIP	SLN 50	50.7 ± 0.1	0.08 ± 0.02	−4.0 ± 0.6	-
	NLC 50	49.2 ± 0.9	0.13 ± 0.01	−7.7 ± 0.3	-
	NLC 25	24.9 ± 0.5	0.17 ± 0.02	−7.4 ± 0.4	-
	NLC 100	119.6 ± 0.7	0.09 ± 0.03	−7.5 ± 0.3	-
+ CIP	SLN 50	50.1 ± 0.3	0.11 ± 0.04	−3.7 ± 0.4	97.9 ± 0.2
	NLC 50	48.9 ± 0.6	0.07 ± 0.01	−7.1 ± 0.7	98.0 ± 0.3
	NLC 25	25.1 ± 0.2	0.21 ± 0.03	−6.2 ± 0.1	80.4 ± 0.4
	NLC 100	107 ± 2	0.20 ± 0.02	−7.4 ± 0.4	98.7 ± 0.2

The mean ± SD of n = 9 from three independent experiments is shown.

**Table 2:** Lumped-Gonzo model parameters for CIP release from lipid-based nanocarriers.

Nanocarrier	*a* (%min^−1^)	*b* (%min^−1^)	R^2^
SLN 50	1.9305	0.0176	0.9878
NLC 50	1.4555	0.0129	0.9978
NLC 25	32.9134	0.3235	0.9968
NLC 100	0.3108	0.0140	0.9912

**Table 3:** Pharmaceutical parameters for CIP release from lipid-based nanocarriers.

Nanocarrier	*t*_15%_ (min)	*MDT*_15%_ (min)	*X*% at 30 min (%)	*DE*_360min_ (%)
SLN 50	9.0	4.3	37.9	75.2
NLC 50	11.9	5.7	31.5	70.8
NLC 25	0.5	0.2	95.6	101.4
NLC 100	148.8	47.6	6.6	14.3

## Data Availability

The original contributions presented in this study are included in the article/Supplementary Material. Further inquiries can be directed to the corresponding author.

## Supplementary Materials

The following supporting information can be downloaded at: https://www.mdpi.com/article/10.3390/pharmaceutics18060727/s1, Figure S1: Particle size distribution of SLN 50 (a), NLC 50 (b), NLC 25 (c) and NLC 100 (d); Figure S2: Histograms of the distribution of diameters and height profiles of SLN 50 (a,b), NLC 50 (c,d), NLC 25 (e,f) and NLC 100 (g,h) using AFM; Figure S3: PdI changes in SLN 50 (a), NLC 50 (b), NLC 25 (c) and NLC 100 (d) at different storage conditions.

## Abbreviations

The following abbreviations are used in this manuscript:

SLN	Solid Lipid Nanoparticles
NLC	Nanostructures Lipid Carriers
CIP	Ciprofloxacin
DLS	Dynamic Light Scattering
TEM	Transmission electron microscopy
AFM	Atomic Force Microscopy
PdI	Polydispersity Index
$E_{ef}$	Encapsulation Efficiency
SD	Standard Deviation
ZP	Zeta Potential
$t_{x\%}$	Release Time
X%*_t_*	Percentage of Drug Released
DE	Dissolution Efficiency
${MDT}_{x\%}$	Mean Dissolution Time
$M_{t}\%$	Percentage of Drug Released at Time t
a	Lumped-Gonzo model representative parameters
b	
