# Supplementary material for: Systematic Study of Ciprofloxacin Release from Lipid-Based Nanocarriers
Source: Pharmaceutics. 2026 Jun 12;18(6):727. doi: 10.3390/pharmaceutics18060727 (PMC13306639; doi:10.3390/pharmaceutics18060727)

Figure S1. Particle size distribution of SLN 50 (a), NLC 50 (b), NLC 25 (c) and NLC 100 (d).

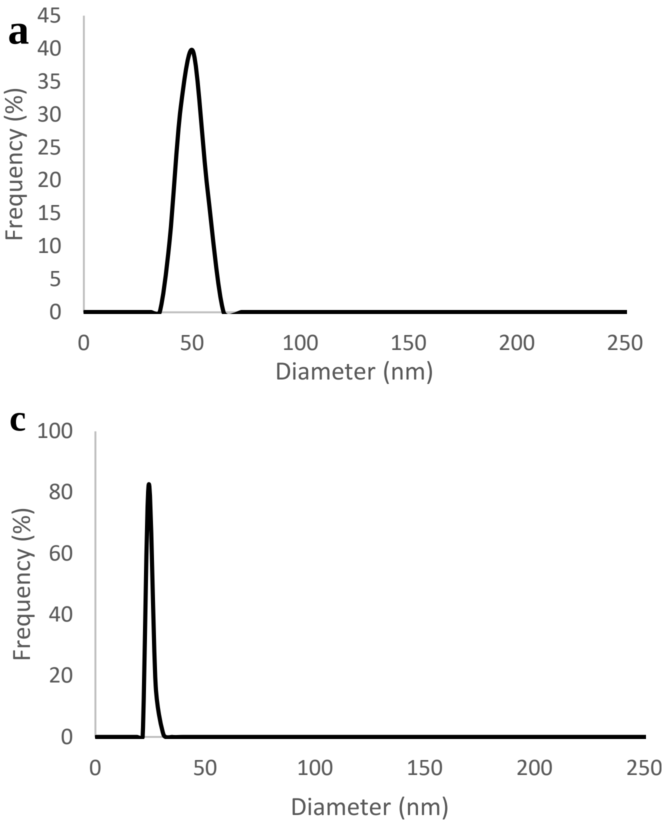

Figure S2. Frequency distribution histograms of nanoparticle lateral diameter and height obtained from AFM image analysis for SLN 50 (a-b), NLC 50 (c-d), NLC 25 (e-f), and NLC 100 (g-h). Mean values are indicated in each histogram. Height corresponds to the vertical dimension (z-axis) measured from the substrate surface in the AFM topographic images.

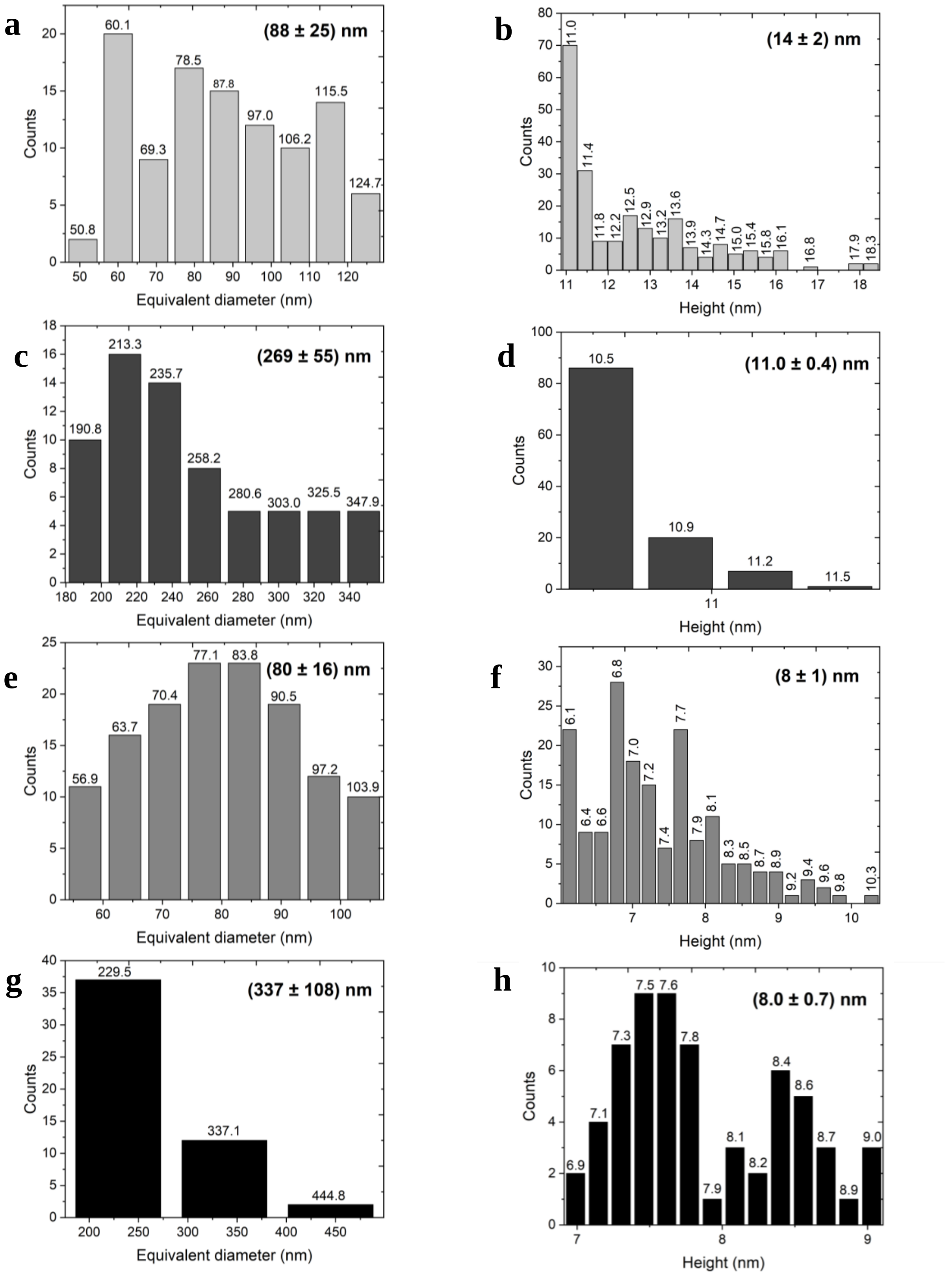

Figure S3. PdI changes of SLN 50 (a), NLC 50 (b), NLC 25 (c) and NLC 100 (d) at different storage conditions.

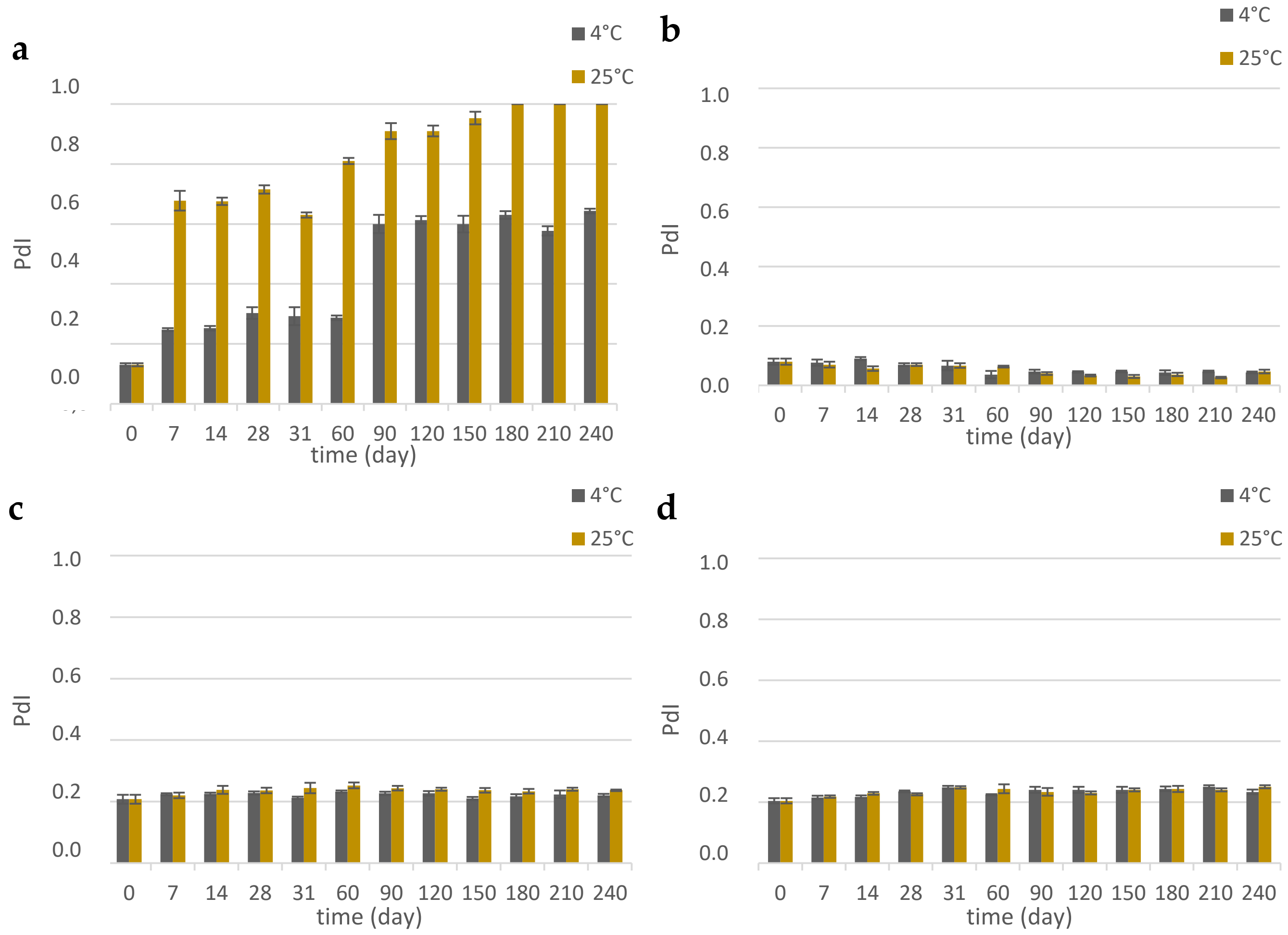

Supplement: Supplementary file 1 [file pharmaceutics-18-00727-s001.zip › pharmaceutics-4324600-supplementary.pdf]
